# Copy Number Variants in Candidate Genes Are Genetic Modifiers of Hirschsprung Disease

**DOI:** 10.1371/journal.pone.0021219

**Published:** 2011-06-21

**Authors:** Qian Jiang, Yen-Yi Ho, Li Hao, Courtney Nichols Berrios, Aravinda Chakravarti

**Affiliations:** Center for Complex Disease Genomics, McKusick-Nathans Institute of Genetic Medicine, Johns Hopkins University School of Medicine, Baltimore, Maryland, United States of America; Kyushu Institute of Technology, Japan

## Abstract

Hirschsprung disease (HSCR) is a neurocristopathy characterized by absence of intramural ganglion cells along variable lengths of the gastrointestinal tract. The HSCR phenotype is highly variable with respect to gender, length of aganglionosis, familiality and the presence of additional anomalies. By molecular genetic analysis, a minimum of 11 neuro-developmental genes (*RET*, *GDNF*, *NRTN*, *SOX10*, *EDNRB*, *EDN3*, *ECE1*, *ZFHX1B*, *PHOX2B*, *KIAA1279, TCF4*) are known to harbor rare, high-penetrance mutations that confer a large risk to the bearer. In addition, two other genes (*RET, NRG1*) harbor common, low-penetrance polymorphisms that contribute only partially to risk and can act as genetic modifiers. To broaden this search, we examined whether a set of 67 proven and candidate HSCR genes harbored additional modifier alleles. In this pilot study, we utilized a custom-designed array CGH with ∼33,000 test probes at an average resolution of ∼185 bp to detect gene-sized or smaller copy number variants (CNVs) within these 67 genes in 18 heterogeneous HSCR patients. Using stringent criteria, we identified CNVs at three loci (*MAPK10, ZFHX1B*, *SOX2*) that are novel, involve regulatory and coding sequences of neuro-developmental genes, and show association with HSCR in combination with other congenital anomalies. Additional CNVs are observed under relaxed criteria. Our research suggests a role for CNVs in HSCR and, importantly, emphasizes the role of variation in regulatory sequences. A much larger study will be necessary both for replication and for identifying the full spectrum of small CNV effects.

## Introduction

Among newborns, the most frequent cause of a functional intestinal obstruction is Hirschsprung disease (HSCR: MIM# 142623), or congenital aganglionosis, a neuro- developmental defect associated with the lack of intramural ganglion cells in the myenteric and sub-mucosal plexuses along varying segments of the gastrointestinal tract [1]. The disorder is classified into short-segment (S-HSCR: aganglionosis up to the upper sigmoid colon), long-segment (L-HSCR: aganglionosis up to the splenic flexure and beyond) and total colonic aganglionosis (TCA) forms. HSCR usually occurs as an isolated trait in ∼70% of cases: the remainder comprises those with a recognized chromosomal abnormality, a recognized syndrome or additional congenital anomalies [2]. This birth defect is not uncommon and shows population incidences of 15, 28 and 21 cases per 100,000 live births among Europeans, Asians and Africans, respectively [1]. The disease has all the imprints of a multifactorial disorder and shows high heritability (81%–100%, depending on the sex of the proband and affected sibling), a large sex-difference (3.9 male: female), a high sibling recurrence risk (200-fold greater than the population) and non-Mendelian inheritance in families [1].

Importantly, HSCR displays a highly variable phenotype with variation in recurrence risk by gender, familiality, segment length of aganglionosis and associated phenotypes [3]. The reasons for much of this variation are largely unknown, although gene discovery has clarified some genotype-phenotype correlations. Numerous molecular genetic studies have identified rare high-penetrance mutations in 11 genes (*RET*, *GDNF*, *NRTN*, *SOX10*, *EDNRB*, *EDN3*, *ECE1*, *ZFHX1B*, *PHOX2B*, *KIAA1279, TCF4*) in HSCR [1], [2]. However, cumulatively, these mutations explain only a minority (<5%) of cases. Additional phenotypic variation is explained by two common low-penetrance polymorphic variants at *RET* [4] and *NRG1* [5], but the vast majority (∼80%) of HSCR heritability is still hidden or missing [6].

One possible reason for the hidden heritability in HSCR is the inadequate study of structural variants, i.e., insertions, deletions, inversions and translocations, of which copy number variants (CNVs) are only one part. Three types of studies suggest that such genomic variants may make an important contribution to HSCR risk. First, early cytogenetic studies identified trisomy 21 (Down Syndrome, DS) as a frequent occurrence in HSCR: it is observed in between 2–10% (average 5%) of cases and is, consequently, 40-fold more common than in the general population of newborns; conversely, ∼0.8% of individuals with DS have congenital aganglionosis [7]. Second, large deletions at 10q11–q21, 13q22–q32 and 2q21–q23 have been identified in HSCR patients with additional anomalies [2]. Third, a survey of statistically significant associations between congenital malformations and non-mosaic, recurrent, single, contiguous autosomal deletions and duplications, detectable by karyotyping identified 13q22–q32 and 17q21 deletions and 17q21–q23 duplications in HSCR [8], [9]. Importantly, each of these cytogenetic findings have clarified the genetics of HSCR: DS-associated HSCR is now known to be partially mediated through the *RET* low-penetrance enhancer polymorphism [10], [11]; the deletions at 10q, 13q and 2q contributed to the positional identification of *RET* [12], *EDNRB* [13] and *ZFHX1B* [14], respectively; and, the 17q locus harbors a novel dosage-sensitive HSCR gene [8], [9].

The role of large genomic mutations in HSCR is not in doubt, but these types of mutations are, nevertheless, rare and invariably deleterious. Modern genomic technologies now allow a comprehensive search for structural variation of all sizes in the human genome. Indeed, smaller structural variants are common in the human genome and have been shown to be responsible for many human traits and diseases [15]–[17]. These genomic variants are an important source of phenotypic diversity since they can directly influence the expression of genes in their vicinity in a dosage-dependent manner [18] and probably also the timing of their expression [19]. Consequently, the smaller structural variants can act as strong genetic modifiers of human disease above and beyond their role as susceptibility mutations and, we hypothesize, they will be an integral part of all multifactorial diseases.

Technically, studies of small structural variants, are difficult and so their roles in disease have been incompletely investigated. In HSCR, two studies failed to detect any structural variants in the *RET*, *GDNF*, *EDN3* and *ZFHX1B* genes in 208 Spanish [20] and 80 German [21] patients with largely isolated HSCR. Both of these studies used the MLPA (multiplex ligation-dependent probe amplification) technique to assess dosage changes in the coding sequence only to conclude that structural variants are uncommon in HSCR [20], [21]. Consequently, we conducted a broader search for functional dosage variants (1) using the array CGH (comparative genomic hybridization) method, (2) scanning both coding and non-coding (regulatory) sequences, and, (3) screening a diverse collection of 18 HSCR patients that varied by recognized risk categories, i.e., gender, familiality, segment length of aganglionosis and associated anomalies. We also screened a large collection of 67 known and well-validated candidate HSCR genes that arose from experimental studies in humans and mice. These include genes identified by human linkage analysis, human association studies, large and recurrent genomic deletions in patients, as well as genes for mouse aganglionosis phenotypes and transcripts dys-regulated in the gastro-intestinal (GI) tracts of *Ret* mouse mutants [22]. Our study revealed three variants, an intronic 3.5 kb deletion in *MAPK10*, a recurrent 1.6 kb exonic duplication in *ZFHX1B*, and a recurrent 600 bp 5′UTR duplication in *SOX2*, that are potential modifiers of HSCR. Interestingly, these modifiers are enriched in those HSCR patients who also have additional anomalies beyond aganglionosis.

## Materials and Methods

### Human samples used in the study

We included a diverse collection of patients that differed by the known categories of risk variation, namely, gender, familiality, segment length of aganglionosis and associated phenotypes. In addition, we did not sample any case that was already known to harbor a structural variant. We sampled 18 Hirschsprung disease patients of whom 16/2 were male/female, 6/12 were multiplex/simplex cases, 8/3/4/3 had the aganglionic segment as S-HSCR/L-HSCR/TCA/unknown, and, 10/8 were isolated/had additional anomalies. The vast majority of our patients, including these 18, are of European origin given our sites of collection but we do not have this information on 9 cases since providing information on race/ethnicity was voluntary. These patients are not a random collection but chosen to represent all categories in a first set of experiments. We studied DNA from the proband (where available) or a sibling with the phenotype of interest. The parental and available family members' DNA of subject 150.3 were also examined for the *MAPK10* deletion to determine its segregation pattern. All patient samples were obtained with written informed consent approved by the Johns Hopkins University School of Medicine IRB. For aCGH studies we purchased control DNA from Promega Corporation (Madison, WI, USA) that included a mixture of genomic DNA from six unrelated males and six unrelated females, respectively.

### Candidate genes selected for aCGH

We opted to include only well-validated HSCR genes, as opposed to suspected pathway-based gene selection, to increase the likelihood of detecting and interpreting structural variants. Consequently, we selected 67 genes for study that were proven to have a role in HSCR by the identification of mutations in human patients (12 genes) or in mouse models of aganglionosis (2 genes), or shown to have statistically significantly altered gene expression in comparisons of the GI tract of *Ret^+/+^* (wild-type) versus *Ret* ^−/−^ (null) mice (53 genes). Specifically, the categories and genes selected were: (1) Genes from human linkage analysis (n = 10): *RET*, *GDNF*, *SOX10*, *EDNRB*, *EDN3*, *ECE1*, *ZFHX1B*, *KIAA1279*, *GRB10, NRTN*; (2) Genes from human association studies (n = 2): *NRG1*, *SEMA3A*; (3) Genes recognized from large recurrent deletions (n = 3): *RET*, *EDNRB*, *ZFHX1B*; (4) Genes recognized through a mouse aganglionosis phenotype (n = 2): *PHACTR4*, *ZIC2*; (5) Genes dys-regulated by mouse *Ret* mutation (n = 53): *ELAVL4*, *SYT11*, *MLLT11*, *TGFB2*, *DLX1*, *HOXD4*, *TMEFF2*, *ARHGEF3*, *TAGLN3*, *GAP43*, *SERPINI1*, *SOX2*, *CRMP1*, *UCHL1*, *PHOX2B*, *MAPK10*, *CARTPT*, *PCDHA1*, *DPYSL3*, *NSG2*, *VIP*, *ETV1*, *STMN2*, *ELAVL2*, *GFRA1*, *HMX3*, *EBF3*, *GNG3*, *PHOX2A*, *CADM1*, *PRPH*, *ASCL1*, *TBX3*, *MAB21L1*, *GNG2*, *SCG3*, *TUBB3*, *MAPT*, *HOXB5*, *CDH2*, *STMN3*, *SOD1*, *IL10RB*, *IFNGR2*, *SON*, *CBR1*, *TTC3*, *TFF3*, *CSTB*, *PFKL*, *DCX*, *FGF13*, *L1CAM*. Detailed information on these HSCR genes and their probe coverage on the CGH array we used, as well as the literature citation demonstrating their candidacy, are provided in Table S1. All gene and locus positions were with respect to the human genome build hg18.

### High-density array CGH (aCGH) design

For aCGH analysis we used the Agilent 4×44 K custom-designed high-density microarray consisting of 45–60 nt (nucleotide) isothermal oligonucleotide probes. Each array consisted of 32,330 test probes and 12,885 control probes (45,215 total probes per array). The test probes covered the 67 HSCR genes from their 5′UTR to their 3′UTR with a higher density of tiling probes across each annotated exon ±20 nt. Thus, each gene was represented at an average resolution of 185 nt but this was ∼25 nt and ∼240 nt for coding and non-coding regions, respectively. The control probes had an average coverage of ∼348 kb and provided a genomic backbone and were of four types: 1,262 Agilent normalization probes, 301×5 Agilent replicate probes, 2,118 Agilent control probes and 8,000 custom control probes.

For hybridization analysis, test DNAs were digested with AluI and RsaI followed by labeling the test DNAs with Cy5-dCTP (red fluorescence) and the sex-matched control DNA with Cy3-dCTP (green fluorescence), using the Invitrogen BioPrime Array CGH genomic labeling kit (Invitrogen Corporation, Carlsbad, CA). Purification of labeling products, array hybridization and washing were performed according to the manufacturers' instructions. The slides were then scanned into image files using an Agilent High-Resolution Microarray Scanner. Quantification of each image file was achieved using the Agilent Feature extraction software (v9.5), and text file outputs were imported into an in-house analysis package for dosage analysis.

### Data Analysis and Structural Variant detection

After image analysis, the raw intensities from the red and green channels were adjusted by subtraction of the background intensity and preprocessed using a variance stabilizing normalization procedure. This procedure, called vsn2, provides straightforward methods for preprocessing two-channel arrays by calibrating the dependency between the mean and variance of the raw intensity measurements while accounting for both the foreground and background intensities, within a model framework [23]. This analysis was performed for each array analyzed. To avoid the influence of outliers in the raw intensities, we trimmed the largest 10% of the residuals when estimating the parameters in vsn2. Consequently, we performed the locally weighted linear regression using the *lowess* function in R [24], for the red and green channels separately to smooth out the systematic effect caused by the GC-content variation in the probe sequences. These final residuals from the *lowess* regression were used for further analysis. To control for batch effects during experimental array processing, we utilized the 1,262 normalization probes from Agilent and our 8,000 control probes. We performed quantile normalization on each array so that the red and green intensities of the control probes had the same mean absolute difference (MAD) and the log_2_ ratio of the red over green intensities had their median centered at zero. The between array normalization procedure was performed using the marray R package available through www.bioconductor.org.

The post-normalization relative intensity data log_2_R, where R =  red/green intensity, were plotted against their genomic location and outliers were smoothed using a sliding window of five probes. For a given smoothed region, we calculated its median (m) from the local observations. We also trimmed the most extreme 2.5% of observations to calculate the standard deviation 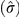 for the entire data set. For a given smoothed region, an outlier was determined and smoothed if the difference between maximum or minimum intensity and its closest neighboring probe exceeded 4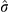: we replaced these observations by m 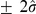. After smoothing of outliers, we used the circular binary segmentation procedure (CBS) [25] to detect copy number with a Type-I error (α) of 0.01. For the segmented region reported by CBS, we examined the intensities across ≥10 probes and used a *stringent* criterion to call a duplication if log_2_R≥ log_2_(1.5)  =  +0.58, a deletion if log_2_R ≤ log_2_(0.5)  =  −1, and a normal diploid dosage if −1< log_2_R <0.58. Although the *stringent* criterion can reduce false positive CNV identification it can also increase the false negative rate. Thus, we also analyzed our results using the following *relaxed* criterion: call a duplication if log_2_R≥ log_2_(1.5)  =  +0.40, a deletion if log_2_R ≤ log_2_(0.5)  =  −0.80, and a normal diploid dosage if −0.80< log_2_R <0.40. Finally, we also used the commercial proprietary software provided by Agilent (Genomic Workbench, Standard Edition, V 5.0.14) to call CNVs. Nevertheless, the actual duplication and deletion calls utilized the identical *stringent* and *relaxed* criteria indicated earlier. The array data have been presented in accordance with MIAME guidelines and deposited in NCBI's Gene Expression Omnibus [26]. All data are accessible through the GEO Series accession number GSE29051 (http://www.ncbi.nlm.nih.gov/geo/query/acc.cgi?acc=GSE29051).

### PCR assays

For further analysis of aCGH-inferred CNVs, we initially used the oligonucleotide intensity data to approximate the genomic breakpoint positions for each variant. For *MAPK10* deletion analysis, based on these coordinates, we designed inward-facing primers to amplify the deletion allele (forward primer 5′ to 3′: TTGACAAGCTCCCACCAACATAAT; reverse primer 5′ to 3′: ACCAGCAACCATGATGAAGTGAAT). Standard PCR was then conducted with Thermo-Start PCR Master Mix (AB-0938/15/DC/B). A 50 µl PCR reaction was performed with 1 µM of each primer, 25 µl of 2×Thermo-Start PCR Master Mix and 50 ng of template DNA. The PCR conditions were as follows: 95°C for 15 min, 35 cycles of 95°C for 20 s, 60°C for 30 s and 72°C for 1 min, followed by 72°C for 5 min.

## Results

We performed analysis on 18 patient samples using 28 arrays, involving replicate samples, to assess the reliability of results. Specifically, 11 samples (#'s 47, 150, 242, 252, 346, 348, 359, 370, 372, 384, 423) were studied once, 4 (#'s 300, 354, 355, 408) were examined twice while 3 (#'s 63, 122, 413) were studied in triplicate. First, in order to investigate the consistency of the intensity readings between the technical replicates, we calculated the Pearson correlation of the raw intensities between replicates (mean r = 0.72 across 13 comparisons) and between randomly selected samples (mean r = 0.42 across 13 comparisons), a difference that was statistically significant (P = 0.004). Second, we investigated whether this difference would be enhanced after statistical pre-processing of arrays. After normalization, the mean correlation was 0.69 between replicates and 0.32 between randomly selected samples, a difference that was more enhanced (P<0.0001). Since the results between technical replicates were similar, we averaged the log_2_R between technical replicates for CNV segmentation analysis.

We next investigated the role of stringent versus relaxed criteria on CNV assessment. Consequently, we identified all CNVs meeting our criteria using our method and Agilent's method for identifying dosage variants. First, none of our analyses identified a single large deletion or duplication involving an entire or the majority of any of the 67 genes we investigated. Consequently, we presume that these are rare in the HSCR population and so we need to screen larger numbers of cases. Our method, however, identified 3 small dosage variants, namely, a deletion in *MAPK10*, a duplication involving *ZFHX1B*, and a duplication involving *SOX2* using the stringent criterion. Under the relaxed criterion, we identified 2 additional deletions of *PHOX2B* and *SEMA3A*, each occurring in two patients. Using the Agilent software, we identified 5 additional duplications (*GDNF*: 2 cases, *GNG2*: 1 case, *TTC3*: 2 cases, *GAP43*: 1 case, *NRG1*: 1 case) but failed to see the prior *SEMA3A* deletion. Thus, the Agilent methods detected almost twice as many CNVs (9) as our method (5), while there was about equal concordance (60%) between the stringent vs. relaxed criterion on both methods. Finally, for the three CNVs common to all methods, the concordance within either method and across stringency was 76% while that between the methods within stringency was 46% (relaxed) to 67% (stringent). Consequently, we used our method and the stringent criterion to identify biologically significant deletions and duplications.

**Figure 1:**
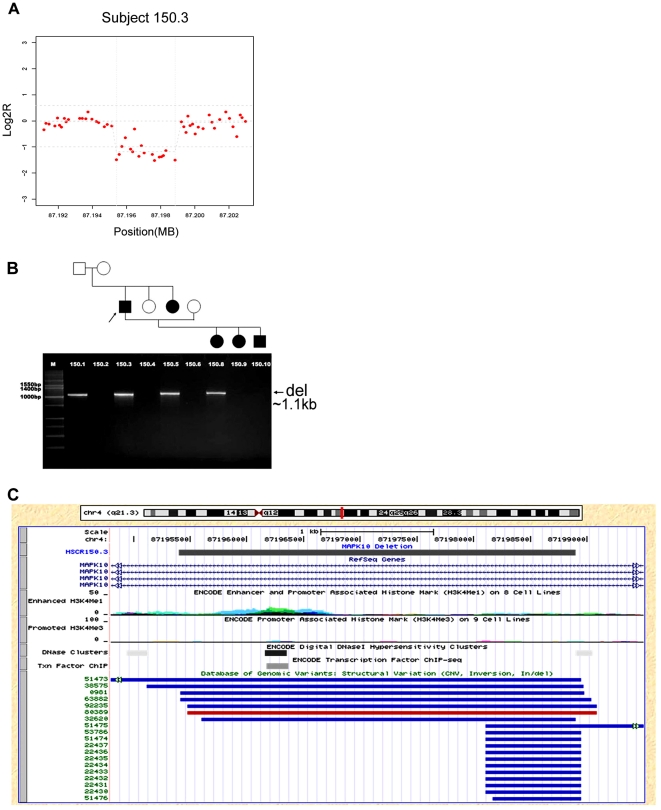
*MAPK10* dosage variant in Hirschsprung disease. We show the *MAPK10* deletion profile from aCGH analysis (A), its segregation pattern within a family (B), and, the corresponding genomic locus in the UCSC Genome Browser (hg18) (C). The grey rectangle in (C) delineates the CNV region in subject 150.3; RefSeq gene boundaries are shown in light blue; the Database of Genomic Variants entries are shown with red indicating gain and blue indicating loss of material relative to the reference sequence.

**Figure 2:**
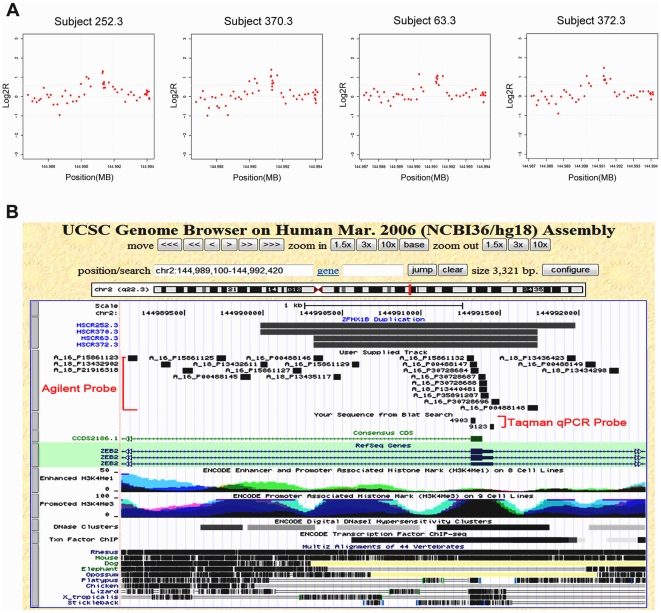
*ZFHX1B* dosage variant in Hirschsprung disease. We show the *ZFHX1B* duplication profile from aCGH analysis (A), and, the corresponding genomic locus in the UCSC Genome Browser (hg18) (B). Functional annotation of this region shows multiple DNase I hypersensitive sites, transcription factor binding sites and promoted H3K4me3 histone marks. Evolutionary conservation across species is also shown.

**Figure 3:**
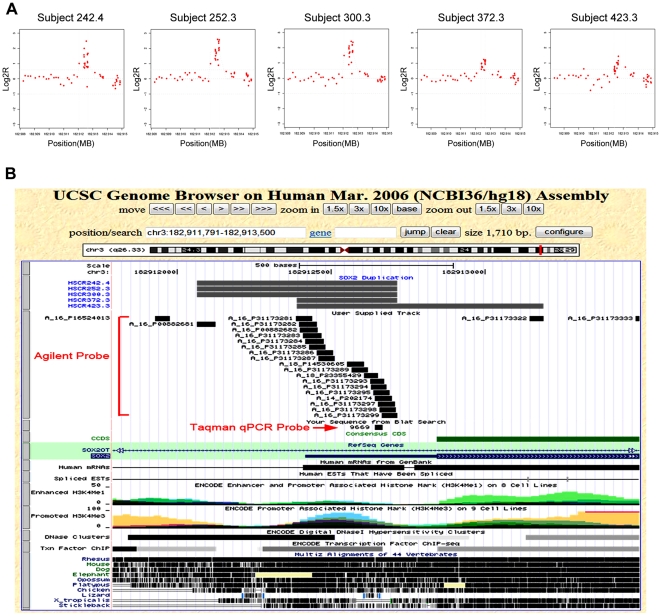
*SOX2* dosage variant in Hirschsprung disease. We show the *SOX2* duplication profile from aCGH analysis (A), and, the corresponding genomic locus in the UCSC Genome Browser (hg18) (B). Functional annotation of this region shows multiple DNase I hypersensitive sites, transcription factor binding sites and promoted H3K4me3 histone marks. Evolutionary conservation across species is also shown.

For further analysis we restricted attention to the three common variants in 18 heterogeneous HSCR patients (Table 1): deletion in intron 11 of *MAPK10*, duplication involving exon 2 of *ZFHX1B*, and duplication in the 5′UTR of *SOX2*. The *MAPK10* deletion, on chromosome 4 at location 87,195,268 (hg18), was a 3.5 kb lesion detected using 13 probes in a single sample (Figure 1A). The *ZFHX1B* duplications, on chromosome 2 and starting at locations 144,989,981–144,990,319 (hg18), were detected in 4 patients using between 10–11 probes and were between 1.42–1.99 kb in length (Figure 2A). Finally, the *SOX2* duplications, on chromosome 3, starting at locations 182,912,064–182,912,387 (hg18), were detected in 5 patients using between 12–19 probes and were between 330–800 bp in length (Figure 3A). The most parsimonious explanation of the *ZFHX1B* and *SOX2* duplications detected in multiple individuals is that it is the same genetic mutation in each gene, with the differences arising from random experimental noise across arrays. The detection of the same CNV in different array experiments and the demonstration of segregation of the solitary *MAPK10* deletion (see below) suggests that these structural variants are biologically meaningful.

**Table 1:** Copy number variants (CNVs) detected by array comparative genomic hybridization (aCGH) in 8 Hirschsprung disease patients.

Subject	Gene	Copy number change	Location(hg18)	Size (kb)	# probes	Mean log_2_R
252.3	*ZFHX1B*	Gain	Chr2: 144,989,981	1.99	10	0.65
370.3	*ZFHX1B*	Gain	Chr2: 144,989,981	1.76	10	0.76
63.3	*ZFHX1B*	Gain	Chr2: 144,990,319	1.42	10	0.95
372.3	*ZFHX1B*	Gain	Chr2: 144,990,319	1.42	11	0.86
242.4	*SOX2*	Gain	Chr3: 182,912,064	0.65	18	1.02
252.3	*SOX2*	Gain	Chr3: 182,912,064	0.65	19	1.84
300.3	*SOX2*	Gain	Chr3: 182,912,064	0.65	18	1.73
372.3	*SOX2*	Gain	Chr3: 182,912,387	0.33	16	0.96
423.3	*SOX2*	Gain	Chr3: 182,912,387	0.80	12	0.73
150.3	*MAPK10*	Loss	Chr4: 87,195,268	3.50	13	−1.29

The main question is whether these three CNVs contribute to the HSCR phenotype. Family studies are one way to assess the biological relevance of a rare variant, as with the *MAPK10* variant. The 3.5 kb *MAPK10* deletion, detected in subject 150.3, was confirmed by a standard PCR assay which amplified only the deletion allele of 1.1 kb; the expected product size of the wild type allele is 4,608 bp but was not observed because the experiment was optimized for smaller fragment only. Segregation analysis of this deletion in related family members showed that the proband inherited this deletion from his unaffected father (the mother is unaffected as well), shares it with a HSCR-affected sister and transmitted it to only one of his three HSCR-affected children (Figure 1B). Under pure autosomal dominant inheritance, it would be unlikely that the *MAPK10* deletion would be a necessary and sufficient explanation of HSCR in this family. This family, however, shows complex inheritance because all five affected family members harbor a *RET* S649S mutation, which although synonymous we have previously demonstrated to lead to aberrant exon 11 splicing and shows strong linkage to a *RET* modifier locus on human chromosome 9q31 [27]. Consequently, we hypothesize that the *MAPK10* deletion is another genetic component to the multifactorial HSCR risk in this family since this mitogen-activated protein kinase 10 gene is highly expressed in the myenteric layer of the intestine and is the most significantly down-regulated gene in the mouse *Ret* ^−/−^ GI tract [22]. Biochemically, the protein acts as an integration point for multiple biochemical signals, and is involved in a wide variety of cellular processes such as proliferation, differentiation, transcription regulation and development. On the other hand, a number of small CNVs within this intron 11 are observed in multiple unrelated healthy controls or Hapmap samples (at frequencies between 2% to 6.6%) [28]–[30] in the Database of Genomic Variants (DGV) (Figure 1C). Although, this would seem to argue against a functional role for the deletion we observed the frequency is small, the allele may only be a susceptibility variant, and, the deletion, along with 5 deletions and 1 duplication found in the DGV, does involve a DNase I hypersensitive site cluster, a transcription factor binding site cluster and an activating H3K4me1 histone mark (Figure1C). These sites overlap significantly and, therefore, could represent the same binding site for MAPK10 activation. Although yet unproven, these results suggest that haplo-insufficiency for this regulatory site may be a modifier for HSCR *per se* or the other associated traits in this patient and his family (Table 2).

**Table 2:** Clinical features, *RET* gene sequence, enhancer genotype and dosage mutations in aCGH analysis of 18 Hirschsprung disease patients.

Subject	Gender	HSCR type	Segment length	Additional anomalies	*RET* mutation	*RET* enhancer variant	CNV
408.3	Male	Isolated	S-HSCR	none	NA	NA	-
346.3	Male	Isolated	L-HSCR	none	none	CT	-
370.3	Male	Isolated	S-HSCR	none	none	CC	*ZFHX1B* duplication
354.3	Male	Isolated	S-HSCR	none	none	CT	-
355.3	Male	Isolated	L-HSCR	none	none	TT	-
359.3	Male	Isolated	S-HSCR	none	none	CC	-
413.3	Male	Isolated	S-HSCR	none	NA	NA	-
384.3	Male	Isolated	S-HSCR	none	none	TT	-
122.7	Male	Isolated	TCA	none	none	TT	-
47.3	Male	Isolated	S-HSCR	none	none	TT	-
348.3	Male	Additional anomaly	L-HSCR	Duane anomaly	none	TT	-
252.3 *	Male	Additional anomaly	TCA	UT reflux, Meckel's diverticulum	L404P	CT	*ZFHX1B* duplication;*SOX2* duplication
300.3	Female	Additional anomaly	unknown	GI malrotation	none	CT	*SOX2* duplication
372.3 *	Male	Additional anomaly	TCA	Neuronal intestinal dysplasia	S307L	CT	*ZFHX1B* duplication;*SOX2* duplication
242.4 *	Female	Additional anomaly	TCA	Ptosis	F998L	CT	*SOX2* duplication
423.3 *	Male	Additional anomaly	S-HSCR	Ptosis	NA	NA	*SOX2* duplication
63.3 *	Male	Additional anomaly	unknown	Strabismus	I464V	TT	*ZFHX1B* duplication
150.3 *	Male	Additional anomaly	unknown	Strabismus	S649S	CC	*MAPK10* deletion

Abbreviations: S-HSCR, short segment HSCR; L-HSCR, long segment HSCR; TCA, total colonic aganglionosis; NA  =  not available.

Patients from multiplex families are indicated by *. *RET* enhancer variant refers to *rs2435357* in intron 1 of *RET*, with ancestral allele.

C and derived mutation allele T.

The recurrent novel duplications of *ZFHX1B* we identified in four patients (Figure 2A) is likely biologically meaningful as well. The aCGH data are internally consistent across replicates to show a small 1.42–1.99 kb duplication involving exon 2 and including part of intron1 (Figure 2B). Nevertheless, two pairs of inward and two pairs of outward primers failed to verify the structural variant (data not shown), although a TaqMan copy number assay (# 9123 in Figure 2B) detected one copy for subject 370.3 and two copies for the other three subjects. These results are not unexpected for a duplication where the presence of additional similar sequence can lead to multiple priming sites beyond the ones intended. Annotations of this region show evolutionary conservation of exon 2 to lizard, *Xenopus tropicalis* and teleost fish (Figure 2B). Functional annotations show multiple DNase I hypersensitive sites, transcription factor binding sites and promoter H3K4me3 histone marks (Figure 2B). These sites overlap significantly with the coding exon 2 and immediately downstream sequences and, therefore, could represent either duplication or disruption of a wild type regulatory element for *ZFHX1B*. Haplo-insufficiency of *ZFHX1B* is the cause of Mowat-Wilson syndrome and we are unaware what a duplication phenotype might be. However, *ZFHX1B* is such a critical regulator of epithelial- mesenchymal transitions (EMT) throughout neural crest development that it is very likely a modifier of HSCR *per se* or the other associated traits with which it is observed (Table 2).

We identified a second novel recurrent duplication in *SOX2* in five patients (Figure 3A) that is similarly biologically meaningful. The aCGH data are internally consistent across replicates to show a small 600 bp duplication likely involving most of the 5′UTR (Figure 3B). Nevertheless, a TaqMan Copy Number Assay (# 9669 in Figure 3B) failed to detect the variant, as before. Annotation of this region shows evolutionary conservation of the 5′UTR to lizard, *Xenopus tropicalis* and chicken. Functional annotations show multiple DNase I hypersensitive sites, transcription factor binding sites and promoted H3K4me3 histone marks (Figure 3B). These sites overlap significantly and suggest a critical regulatory element for *SOX2* that is disrupted in HSCR and could act as a modifier (Table 2).

Our results, summarized in Table 2, demonstrate a striking feature: the incidence of structural variants in the three genes discovered is higher in the HSCR families with additional anomalies and, indeed, they co-occur in some families. This association is highly significant since the 10 isolated HSCR patients have only one variant but the 8 HSCR cases with additional anomalies have 7 variants (P = 0.0029). If we included all other CNVs detected, specifically the 9 variants detected by Agilent analysis using the relaxed criterion, this association is still highly significant, with the 10 isolated HSCR patients having only two variants but the 8 HSCR cases with additional anomalies having 7 variants (P = 0.015). In other words, the increased frequency of dosage variants is enhanced in the HSCR cases with additional anomalies (except for family #348) and in only one family with isolated HSCR (family #370). Interestingly, the families with multiple anomalies also show a greater frequency of *RET* coding or splicing mutations and these families tend to have more severe (L-HSCR, TCA) forms of HSCR and more *RET* enhancer variant heterozygotes, as previously noted in trisomy 21-HSCR cases [11]. These data suggests two classes of HSCR patients. The families which have anomalies in addition to HSCR, none of which have recognized syndromes (such as Down, Mowat-Wilson, Shah-Waardenburg, etc.), have a propensity to be familial and harbor multiple mutations both in the major gene *RET* and in *MAPK10*, *ZFHX1B*, and *SOX2.* On the other hand, most patients with isolated HSCR are neither familial nor have *RET* coding or structural variant mutations. As we have shown elsewhere, and in Table 2, these latter patients are more likely to harbor regulatory polymorphisms such as in the *RET* intron 1 enhancer [3], [4] or intron 1 of *NRG1* [5].

## Discussion

HSCR is a multifactorial disorder where multiple rare and common mutations exist within each patient. With the exception of the major gene *RET*, no other mutation is yet known to be necessary for this neuro-developmental birth defect [1]–[3]. Consequently, any single HSCR gene is expected to be mutant in only a subset of patients implying that the number of genes involved and their mutational types are numerous. In this study, we conducted an investigation into the frequency of small structural variants in HSCR since they are numerous in the human genome [15]–[17] and likely to exist in all genes, including in HSCR genes. Studies by others have assessed the role of these small CNVs in 288 European patients, but in only 4 HSCR genes [20], [21]. We chose instead to search for such dosage mutations in a more extensive set of 67 HSCR genes, all involved in enteric nervous system (ENS) development, in 18 diverse HSCR patients. We identified no changes that were full gene deletions or duplications although we had the ability to detect such mutations and specifically included cases with multiple anomalies where this would have been a likely outcome. Therefore, like others [20], [21], we conclude that the frequency of whole gene dosage mutations are rare in HSCR. However, three variants in *MAPK10*, *ZFHX1B*, and *SOX2*, that are all likely regulatory, but of unknown specific function, were identified in 8 out of 18 patients (44%) and appeared to be modifiers of HSCR. These three genes point to pathways of critical importance to ENS development, supporting that their disruption may lead to HSCR.

*MAPK10* encodes a neuronal-specific form of c-Jun N-terminal kinases (JNKs) and is highly expressed in the mouse myenteric segment of the intestine [22]. In the mouse, Mapk10 activation is associated with responses to inflammation and cellular stresses, but also with cytoskeletal changes associated with neuronal growth. Mapk10 binds and phosphorylates Stmn2 (Stathmin like-2) regulating its microtubule-destabilizing activity [22]. Since *Ret*, *Mapk10*, and *Stmn2* are all expressed in the ENS, we hypothesize that these molecules function together to link extracellular signals to the rearrangement of the neuronal cytoskeleton required for axonal outgrowth [31]. As explained earlier, the deletion variant is most likely a regulatory mutation that deletes a dosage-dependent binding site for MAPK10 activation. This is a rare polymorphism in humans (2% to 6.6%) [28]–[30] and probably has no effect on its own but could lead to HSCR in the context of a *RET* splicing mutation (S649S) that this patient also harbors.

A critical step in neural crest cell genesis is the developmental transition of neuro-epithelial cells to a mesenschymal fate (EMT), a transition strongly regulated by two related proteins ZEB1 and ZEB2 (the protein encoded by *ZFHX1B*) [32], [33]. These proteins act so early in neural crest development that it is not surprising that *de novo* heterozygous deletions of *ZFHX1B* lead to HSCR-related Mowat-Wilson syndrome. The protein encoded by this gene is a member of the Zfh1 family of 2-handed zinc finger/homeodomain proteins. It is located in the nucleus and functions as a DNA-binding transcriptional repressor that interacts with activated SMADs. It is difficult to predict what duplication of exon 2 might do, but duplications usually lead to milder phenotypes. Since all four patients with *ZFHX1B* duplications also carry a *RET* mutation we suspect that these duplications act as a HSCR modifier. A broader search for *ZFHX1B* regulatory mutations in HSCR would clarify this relationship with RET signaling.

*SOX2* is an intronless gene that encodes a member of the SRY-related HMG-box family of transcription factors involved in the regulation of embryonic development and in the determination of cell fate. The protein is strongly implicated in the determination of neurogenesis and also regulates gene expression in the stomach; it is expressed in the mouse ENS in a pattern indistinguishable from that of the established ENS transcription factors *Mash1*, *Phox2a*, *Phox2b* and *Sox10* [22]. In fact, *SOX1-3* keep neural cells undifferentiated by counteracting the activity of proneural proteins and the generation of neurons from stem cells critically depends on the inhibition of *SOX1-3* expression [34], [35]. Consequently, we hypothesize that duplications involving the 5′UTR of this gene might lead to increased expression of *SOX2* and affect the process of neurogenesis by maintaining proliferation and/or the maintenance of neural stem cells. This can lead to modification of ganglionosis, and thus HSCR. Interestingly, 3 of 4 SOX2 duplication carriers also carried *RET* mutations suggesting another basis for interaction.

Our research suggests a role for CNVs in HSCR and, importantly, emphasizes the role of variation in regulatory sequences. Most studies of structural variation in human disease, based on array CGH, focus on large lesions of 300 kb or greater. This has become a powerful tool for the molecular elucidation and diagnosis of disorders resulting from genomic copy number variation. However, the vast majority of intragenic deletions or duplications, that is smaller than a gene, have remained beyond the detection limit of most clinical aCGH analyses. The primary reasons for this are the increased numbers of probes needed for genomic resolution, and the consequent higher cost, the difficulty of interpreting non-coding CNVs, and the enhanced detection of benign CNVs that can confound clinical interpretation. However, the increasing biological annotation of the non-coding segments of the human genome [36] is improving the interpretation of non-coding CNVs [37]–[39].

This study raises two major questions in human medical genetics with respect to causation, namely, (1) which genes should be considered primary (main) and which modifiers, and, (2) which patients are most likely to harbor multiple gene or genomic mutations? The accepted definition of a modifier gene (allele) is one that is not necessary for disease causation (principal gene) but one that alter phenotype penetrance and expressivity. Consequently, the role of modifiers in the inheritance of single gene disorders is clear, as first suggested by Haldane [40]. For complex multifactorial disorders, such as HSCR, this definition can be extended to the multiple principal genes that impart risk by coordinate action and the multiple modifier genes that modulate their response. In this case, since none of the principal genes cause disease by themselves alone they are individually susceptibility genes. In reality, no gene can be precisely classified as principal, modifier or susceptibility since a gene may impart different effects and have different roles depending on the mutation it harbors. In HSCR, the 11 neuro-development genes with confirmed mutations are all susceptibility genes since they affect disease risk but not with complete penetrance suggesting the action of other genes. However, *RET* should be considered principal since nearly all HSCR patients have at least one loss-of-function *RET* mutation. In this sense, *MAPK10*, *ZFHX1B*, and *SOX2* CNVs co-occur with *RET* mutations and given their biology is postulated to further antagonize *RET* function and are, thus, modifiers. But, these genes are not universal modifiers since they are rare in patients without loss-of-function mutations in *RET.* HSCR in this latter group of patients probably occur by different, as yet, unknown mechanisms. These arguments also suggest that the search for modifiers is better restricted to patients with severe mutations and additional anomalies.

This study is the first systematic investigation of gene-sized or smaller structural variants in a large number of HSCR genes in a cohort of isolated and additional anomalies HSCR patients using aCGH analysis. Our experiments show the value of documenting these small structural variants which likely act as modifiers of additional anomalies in HSCR and begin to explain the multifactorial inheritance of this common ENS developmental defect. There has been great attention provided to large genomic lesions that demonstrate, like other human mutations, less-than-complete penetrance or the requirement of multiple hits to induce a phenotype [15], [41]. The results in this paper suggest that the same phenomenon likely acts, to a greater extent, with intragenic dosage mutations. These observations beg the question: what is a susceptibility allele and what is a modifier? In this study we have referred to these dosage alleles as modifiers of HSCR since *RET* is necessary for onset of HSCR [3]. We believe that future investigations using exon-based whole genome arrays can identify the full spectrum of such small CNV effects in HSCR.

## Supporting Information

Table S1 Hirschsprung Disease Candidate Gene Summaries. Each entry shows the symbol, MIM accession #, locus, name, function, size, source and citation for each gene chosen for analysis.
(DOC)
