# Supplementary material for: Copy Number Variants in Candidate Genes Are Genetic Modifiers of Hirschsprung Disease
Source: PLoS One. 2011 Jun 21;6(6):e21219. doi: 10.1371/journal.pone.0021219 (PMC3119685; doi:10.1371/journal.pone.0021219)
Supplement: Table S1 — Hirschsprung Disease Candidate Gene Summaries. Each entry shows the symbol, MIM accession #, locus, name, function, size, source and citation for each gene chosen for analysis. (DOC) [file pone.0021219.s001.doc]

Supplementary Table S1: Hirschsprung Disease Candidate Gene Summaries.

| *Gene* | *Locus* | *Gene name* | *Gene function* | *5’-3’UTR gene size (kb)*  *(# test probes)* | *Source* | *Reference* |
| --- | --- | --- | --- | --- | --- | --- |
| *ARHGEF3*  (MIM 612115) | 3p21-p13 | Rho guanine nucleotide exchange factor (GEF) 3 | activates two Rho GTPase family members (RHOA, RHOB) | 351 (1,400) | Expression |  |
| *ASCL1*  (MIM 100790) | 12q23.2 | achaete-scute complex homolog 1 | transcription factor; plays a role in neuronal commitment and differentiation | 2.8 (102) | Expression |  |
| *CADM1*  (MIM 605686) | 11q23.2 | cell adhesion molecule 1 | cell adhesion molecule | 330 (1,692) | Expression |  |
| *CARTPT*  (MIM 602606) | 5q13.2 | CART prepropeptide | encodes a secreted protein which can be converted to smaller, biologically active peptides | 1.8 (34) | Expression |  |
| *CBR1*  (MIM 114830) | 21q22.13 | carbonyl reductase 1 | one of several monomeric, NADPH-dependent oxidoreductases | 3.1 (45) | Expression |  |
| *CDH2*  (MIM 114020) | 18q11.2 | cadherin 2, type 1, N-cadherin (neuronal) | encodes a calcium dependent cell-cell adhesion glycoprotein | 226 (1,124) | Expression |  |
| *CRMP1*  (MIM 602462) | 4p16.1 | collapsin response mediator protein 1 | encodes a cytosolic phosphoprotein family member involved in the semaphorin signal transduction pathway | 72 (349) | Expression |  |
| *CSTB*  (MIM 601145) | 21q22.3 | cystatin B (stefin B) | encodes a stefin that functions as an intracellular thiol protease inhibitor | 2.4 (20) | Expression |  |
| *DCX*  (MIM 300121) | Xq22.3-q23 | doublecortin | encodes a cytoplasmic protein which directs neuronal migration by regulating the organization and stability of microtubules | 118 (489) | Expression |  |
| *DLX1*  (MIM 600029) | 2q32 | distal-less homeobox 1 | homeobox-containing transcription factor | 4.2 (105) | Expression |  |
| *DPYSL3*  (MIM 601168) | 5q32 | dihydropyrimidinase-like 3 | collapsin response mediator family member | 119 (528) | Expression |  |
| *EBF3*  (MIM 607407) | 10q26.3 | early B-cell factor 3 | transcription factor regulating neurogenesis and differentiation | 128 (876) | Expression |  |
| *ECE1*  (MIM 600423) | 1p36.1 | endothelin converting enzyme 1 | involved in proteolytic processing of endothelin precursors (EDN1, EDN 2, EDN 3) to biologically active peptides | 128 (508) | Linkage |  |
| *EDN3*  (MIM 131242) | 20q13.2-q13.3 | endothelin 3 | member of the endothelin family; interaction of this endothelin with EDNRB is essential for development of the ENS | 25 (227) | Linkage |  |
| *EDNRB*  (MIM 131244) | 13q22 | endothelin receptor type B | G protein-coupled receptor activating a phosphatidylinositol-calcium second messenger system | 80 (461) | Linkage,  CNV |  |
| *ELAVL2*  (MIM 601673) | 9p21 | ELAV (embryonic lethal, abnormal vision, Drosophila)-like 2 (Hu antigen B) | neural-specific RNA-binding protein; markers of early differentiating neurons | 136 (813) | Expression |  |
| *ELAVL4*  (MIM 168360) | 1p34 | embryonic lethal, abnormal vision, Drosophila-like 4 (Hu antigen D) | neuronal-specific binding for 3’-uridylate-rich UTR of growth factor mRNAs | 154 (747) | Expression |  |
| *ETV1*  (MIM 600541) | 7p21.3 | ets variant 1 | transcription factor gene | 100 (774) | Expression |  |
| *FGF13*  (MIM 300070) | Xq26.3 | fibroblast growth factor 13 | member of the fibroblast growth factor (FGF) family; involved in a variety of biological processes, including embryonic development | 573 (1,799) | Expression |  |
| *GAP43*  (MIM 162060) | 3q13.1-q13.2 | growth associated protein 43 | encodes a protein that regulates the growth of neuronal axons and modulates the formation of new connections | 98 (482) | Expression |  |
| *GDNF*  (MIM 600837) | 5p13.1-p12 | glial cell derived neurotrophic factor | encodes a highly conserved neurotrophic factor | 27 (158) | Linkage |  |
| *GFRA1*  (MIM 601496) | 10q26.11 | GDNF family receptor alpha 1 | encodes a glycosylphosphatidylinositol(GPI)- linked cell surface receptor for GDNF and NTN | 216 (1,078) | Expression |  |
| *GNG2*  (MIM 606981) | 14q21 | guanine nucleotide binding protein (G protein), gamma 2 | gamma subunit of the G protein, primarily mediates the specificity of a G protein- receptor interaction | 109 (600) | Expression |  |
| *GNG3*  (MIM 608941) | 11p11 | guanine nucleotide binding protein (G protein), gamma 3 | gamma subunit of G proteins; usually contributes to the specificity of the hundreds of receptor signaling pathways involving G proteins | 1.6 (37) | Expression |  |
| *GRB10*  (MIM 601523) | 7p12.2 | growth factor receptor-bound protein 10 | encodes an adapter protein interacting with a number of receptor tyrosine kinases and signaling molecules | 203 (1,003) | Linkage |  |
| *HMX3*  (MIM 613380) | 10q26.13 | H6 family homeobox 3 | homeobox gene family involved in development | 1.7 (17) | Expression |  |
| *HOXB5*  (MIM 142960) | 17q21.3 | homeobox B5 | encodes a nuclear protein with a homeobox DNA-binding domain; functions as a sequence-specific transcription factor involved in lung and gut development | 2.5 (68) | Expression |  |
| *HOXD4*  (MIM 142981) | 2q31.1 | homeobox D4 | homeobox family gene | 1.8 (51) | Expression |  |
| *IFNGR2*  (MIM 147569) | 21q22.11 | interferon gamma receptor 2; (interferon gamma transducer1) | encodes the non-ligand-binding beta chain of the gamma interferon receptor | 34 (180) | Expression |  |
| *IL10RB*  (MIM 123889) | 21q22.11 | interleukin 10 receptor, beta | cytokine receptor family | 31 (141) | Expression |  |
| *KIAA1279*  (MIM 609367) | 10q22.1 | KIAA1279 | kinesin family member 1 binding protein | 28 (220) | Linkage |  |
| *L1CAM*  (MIM 308840) | Xq28 | L1 cell adhesion molecule | encodes an axonal glycoprotein belonging to the immunoglobulin supergene family | 14 (114) | Expression |  |
| *MAB21L1*  (MIM 601280) | 13q13 | mab-21-like 1 (C. elegans) | similar to the MAB-21 cell fate-determining gene found in *C. elegans* | 2.9 (180) | Expression |  |
| *MAPK10*  (MIM 602897) | 4q22.1-q23 | mitogen-activated protein kinase 10 | encodes a member of the MAP kinase family; acts as an integration point for multiple biochemical signals | 438 (1,453) | Expression |  |
| *MAPT*  (MIM 157140) | 17q21.1 | microtubule-associated protein tau | microtubule-associated protein tau | 134 (739) | Expression |  |
| *MLLT11*  (MIM 604684) | 1q21 | myeloid/lymphoid or mixed-lineage leukemia (tri-thorax homolog *Drosophila*); translocated to, 11 | translocation partner of *MLL* | 8.8 (152) | Expression |  |
| *NRG1*  (MIM 142445) | 8p12 | neuregulin 1 | signaling protein that mediates cell-cell interactions and plays critical roles in the growth and development of multiple organ systems | 216 (1,238) | Association |  |
| *NRTN*  (MIM 602018) | 19p13.3 | neurturin | member of the TGF-beta subfamily; signals through RET and GFRA1 to promote survival of neuronal populations | 4.5 (29) | Linkage |  |
| *NSG2 (HMP19)*  *(MIM not available)* | 5q35.2 | neuron-specific protein family member 2 | unknown | 63 (332) | Expression |  |
| *PCDHA1*  (MIM 606307) | 5q31 | protocadherin alpha cluster | encodes neural adhesion proteins which play a critical role in the establishment and function of specific cell-cell connections | 226 (962) | Expression |  |
| *PFKL*  (MIM 171860) | 21q22.3 | phosphofructokinase, liver | encodes the liver subunit of a tetrameric enzyme that catalyzes a key step in glycolysis | 27 (184) | Expression |  |
| *PHACTR4*  (MIM 608726) | 1p35.3 | phosphatase and actin regulator 4 | uncharacterized actin and protein phosphatase 1 (PP1) regulator family member | 131 (498) | Mouse |  |
| *PHOX2A*  (MIM 602753) | 11q13.2 | paired-like homeobox 2a | encodes a protein containing a paired-like homeodomain | 5.1 (45) | Expression |  |
| *PHOX2B*  (MIM 603851) | 4p12 | paired-like homeobox 2b | encodes a member of the paired family of homeobox DNA-associated protein; functions as a transcription factor | 4.9 (162) | Expression |  |
| *PRPH*  (MIM 170710) | 12q12-q13 | peripherin | encodes a cytoskeletal protein found in neurons of the peripheral nervous system | 3.6 (57) | Expression |  |
| *RET*  (MIM 164761) | 10q11.2 | ret proto-oncogene | encodes a receptor tyrosine kinases critical for neural crest cell and ENS development | 53 (517) | Linkage  CNV |  |
| *SCG3*  (MIM 611796) | 15q21 | secretogranin III | a member of the chromogranin/secretogranin family of neuroendocrine secretory proteins | 40 (290) | Expression |  |
| *SEMA3A*  (MIM 603961) | 7p12.1 | sema domain, immunoglobulin domain (Ig), short basic domain, secreted, (semaphorin) 3A | semaphorin family member, important for normal neuronal pattern development | 236 (1,324) | Association |  |
| *SERPINI1*  (MIM 602445) | 3q26.1 | serpin peptidase inhibitor, clade I (neuroserpin), member 1 | encodes a member of the serpin superfamily of serine proteinase inhibitors, which plays a role in the regulation of axonal growth and the development of synaptic plasticity | 90 (429) | Expression |  |
| *SOD1*  (MIM 147450) | 21q22.11 | superoxide dismutase 1 | encodes protein binding copper and zinc ions | 9.3 (74) | Expression |  |
| *SON*  (MIM 182465) | 21q22.11 | SON DNA binding protein | encodes a DNA-binding structural protein | 34 (479) | Expression |  |
| *SOX2*  (MIM 184429) | 3q26.3-q27 | SRY (sex determining region Y)-box 2 | encodes a member of the SRY-related HMG-box (SOX) family of transcription factors involved in the regulation of embryonic development and determination of cell fate | 2.5 (89) | Expression |  |
| *SOX10*  (MIM 602229) | 22q13.1 | SRY (sex determining region Y)-box 10 | encodes a member of the SOX (SRY-related HMG-box) family of transcription factors involved in the regulation of embryonic development and determination of cell fate | 12.2 (124) | Linkage |  |
| *STMN2*  (MIM 600621) | 8q21.13 | stathmin-like 2 | encodes a neuronal growth-associated protein | 55 (252) | Expression |  |
| *STMN3*  (MIM 608362) | 20q13.3 | stathmin-like 3 | encodes the stathmin/oncoprotein 18 family of microtubule-destabilizing phosphoproteins | 13.7 (36) | Expression |  |
| *SYT11*  (MIM 608741) | 1q21.2 | synaptotagmin XI | encodes a synapse-associated protein | 25.7 (294) | Expression |  |
| *TAGLN3*  (MIM 607953) | 3q13.2 | transgelin 3 | encodes a cytoskeleton-associated protein | 15.2 (128) | Expression |  |
| *TBX3*  (MIM 601621) | 12q24.1 | T-box 3 | encodes a transcription factor involved in the regulation of developmental processes | 13.9 (270) | Expression |  |
| *TFF3*  (MIM 600633) | 21q22.3 | trefoil factor 3 (intestinal) | member of the trefoil family; stable secretory proteins expressed in gastrointestinal mucosa | 3.9 (36) | Expression |  |
| *TGFB2*  (MIM 190220) | 1q41 | transforming growth factor, beta 2 | multifunctional cytokine related to morphogenesis, cell differentiation, proliferation and migration | 99 (551) | Expression |  |
| *TMEFF2*  (MIM 605734) | 2q32.3 | transmembrane protein with EGF-like and two follistatin-like domains 2 | encodes transmembrane protein involved in signal transduction | 245 (1,080) | Expression |  |
| *TTC3*  (MIM 602259) | 21q22.2 | tetratricopeptide (TPR) repeat domain 3 | encodes a protein containing 3 TPR motifs; an Akt-specific E3 ligase | 130 (578) | Expression |  |
| *TUBB3*  (MIM 602661) | 16q24.3 | tubulin, beta 3 | encodes a microtubule-associated protein | 14 (53) | Expression |  |
| *UCHL1*  (MIM 191342) | 4p14 | ubiquitin carboxyl-terminal esterase L1 | encodes a peptidase C12 family protein specifically expressed in neurons | 11.5 (100) | Expression |  |
| *VIP*  (MIM 192320) | 6q25 | vasoactive intestinal peptide | member of the glucagon-secretin family | 9.0 (129) | Expression |  |
| *ZFHX1B*  (MIM 605802) | 2q22.3 | zinc finger E-box binding homeobox 2 | transcriptional corepressor of *Smads* | 136 (856) | Linkage,  CNV |  |
| *ZIC2*  (MIM 603073) | 13q32 | Zic family member 2 (odd-paired homolog, Drosophila) | encodes a member of the ZIC family of C2H2-type zinc finger proteins; functions as a transcriptional repressor | 4.7 (73) | Mouse |  |

**References**

1. Thiesen S, Kubart S, Ropers HH, Nothwang HG (2000) Isolation of two novel human RhoGEFs, ARHGEF3 and ARHGEF4, in 3p13-21 and 2q22. Biochem Biophys Res Commun 273: 364-369.

2. Heanue TA, Pachnis V (2006) Expression profiling the developing mammalian enteric nervous system identifies marker and candidate Hirschsprung disease genes. Proc Natl Acad Sci U S A 103: 6919-6924.

3. Huang HS, Kubish GM, Redmond TM, Turner DL, Thompson RC, et al. (2010) Direct transcriptional induction of Gadd45gamma by Ascl1 during neuronal differentiation. Mol Cell Neurosci 44: 282-296.

4. Hagiyama M, Ichiyanagi N, Kimura KB, Murakami Y, Ito A (2009) Expression of a soluble isoform of cell adhesion molecule 1 in the brain and its involvement in directional neurite outgrowth. Am J Pathol 174: 2278-2289.

5. Sultan M, Piccini I, Balzereit D, Herwig R, Saran NG, et al. (2007) Gene expression variation in Down's syndrome mice allows prioritization of candidate genes. Genome Biol 8: R91.

6. Hermiston ML, Gordon JI (1995) Inflammatory bowel disease and adenomas in mice expressing a dominant negative N-cadherin. Science 270: 1203-1207.

7. Schmidt EF, Shim SO, Strittmatter SM (2008) Release of MICAL autoinhibition by semaphorin-plexin signaling promotes interaction with collapsin response mediator protein. J Neurosci 28: 2287-2297.

8. Brannvall K, Hjelm H, Korhonen L, Lahtinen U, Lehesjoki AE, et al. (2003) Cystatin-B is expressed by neural stem cells and by differentiated neurons and astrocytes. Biochem Biophys Res Commun 308: 369-374.

9. Simeone A, Acampora D, Pannese M, D'Esposito M, Stornaiuolo A, et al. (1994) Cloning and characterization of two members of the vertebrate Dlx gene family. Proc Natl Acad Sci U S A 91: 2250-2254.

10. Cobos I, Borello U, Rubenstein JL (2007) Dlx transcription factors promote migration through repression of axon and dendrite growth. Neuron 54: 873-888.

11. Hamajima N, Matsuda K, Sakata S, Tamaki N, Sasaki M, et al. (1996) A novel gene family defined by human dihydropyrimidinase and three related proteins with differential tissue distribution. Gene 180: 157-163.

12. Zardo G, Tiirikainen MI, Hong C, Misra A, Feuerstein BG, et al. (2002) Integrated genomic and epigenomic analyses pinpoint biallelic gene inactivation in tumors. Nat Genet 32: 453-458.

13. Hofstra RM, Valdenaire O, Arch E, Osinga J, Kroes H, et al. (1999) A loss-of-function mutation in the endothelin-converting enzyme 1 (ECE-1) associated with Hirschsprung disease, cardiac defects, and autonomic dysfunction. Am J Hum Genet 64: 304-308.

14. Bidaud C, Salomon R, Edery P, Van Camp G, Pelet A, et al. (1997) [Mutations of the endothelin-3 gene in isolated and syndromic forms of Hirschsprung disease]. Gastroenterol Clin Biol 21: 548-554.

15. Sanchez-Mejias A, Fernandez RM, Lopez-Alonso M, Antinolo G, Borrego S (2009) Contribution of RET, NTRK3 and EDN3 to the expression of Hirschsprung disease in a multiplex family. J Med Genet 46: 862-864.

16. Carrasquillo MM, McCallion AS, Puffenberger EG, Kashuk CS, Nouri N, et al. (2002) Genome-wide association study and mouse model identify interaction between RET and EDNRB pathways in Hirschsprung disease. Nat Genet 32: 237-244.

17. Kenny SE, Hofstra RM, Buys CH, Vaillant CR, Lloyd DA, et al. (2000) Reduced endothelin-3 expression in sporadic Hirschsprung disease. Br J Surg 87: 580-585.

18. Han J, Knops JF, Longshore JW, King PH (1996) Localization of human elav-like neuronal protein 1 (Hel-N1) on chromosome 9p21 by chromosome microdissection polymerase chain reaction and fluorescence in situ hybridization. Genomics 36: 189-191.

19. Akamatsu W, Fujihara H, Mitsuhashi T, Yano M, Shibata S, et al. (2005) The RNA-binding protein HuD regulates neuronal cell identity and maturation. Proc Natl Acad Sci U S A 102: 4625-4630.

20. Flames N, Hobert O (2009) Gene regulatory logic of dopamine neuron differentiation. Nature 458: 885-889.

21. Smallwood PM, Munoz-Sanjuan I, Tong P, Macke JP, Hendry SH, et al. (1996) Fibroblast growth factor (FGF) homologous factors: new members of the FGF family implicated in nervous system development. Proc Natl Acad Sci U S A 93: 9850-9857.

22. Strittmatter SM, Fankhauser C, Huang PL, Mashimo H, Fishman MC (1995) Neuronal pathfinding is abnormal in mice lacking the neuronal growth cone protein GAP-43. Cell 80: 445-452.

23. Salomon R, Attie T, Pelet A, Bidaud C, Eng C, et al. (1996) Germline mutations of the RET ligand GDNF are not sufficient to cause Hirschsprung disease. Nat Genet 14: 345-347.

24. Ivanchuk SM, Myers SM, Eng C, Mulligan LM (1996) De novo mutation of GDNF, ligand for the RET/GDNFR-alpha receptor complex, in Hirschsprung disease. Hum Mol Genet 5: 2023-2026.

25. Angrist M, Bolk S, Bentley K, Nallasamy S, Halushka MK, et al. (1998) Genomic structure of the gene for the SH2 and pleckstrin homology domain-containing protein GRB10 and evaluation of its role in Hirschsprung disease. Oncogene 17: 3065-3070.

26. Fernandez RM, Ruiz-Ferrer M, Lopez-Alonso M, Antinolo G, Borrego S (2008) Polymorphisms in the genes encoding the 4 RET ligands, GDNF, NTN, ARTN, PSPN, and susceptibility to Hirschsprung disease. J Pediatr Surg 43: 2042-2047.

27. Myers SM, Salomon R, Goessling A, Pelet A, Eng C, et al. (1999) Investigation of germline GFR alpha-1 mutations in Hirschsprung disease. J Med Genet 36: 217-220.

28. Hurowitz EH, Melnyk JM, Chen YJ, Kouros-Mehr H, Simon MI, et al. (2000) Genomic characterization of the human heterotrimeric G protein alpha, beta, and gamma subunit genes. DNA Res 7: 111-120.

29. Bober E, Baum C, Braun T, Arnold HH (1994) A novel NK-related mouse homeobox gene: expression in central and peripheral nervous structures during embryonic development. Dev Biol 162: 288-303.

30. Fu M, Lui VC, Sham MH, Cheung AN, Tam PK (2003) HOXB5 expression is spatially and temporarily regulated in human embryonic gut during neural crest cell colonization and differentiation of enteric neuroblasts. Dev Dyn 228: 1-10.

31. Mavilio F, Simeone A, Giampaolo A, Faiella A, Zappavigna V, et al. (1986) Differential and stage-related expression in embryonic tissues of a new human homoeobox gene. Nature 324: 664-668.

32. Murai M, Turovskaya O, Kim G, Madan R, Karp CL, et al. (2009) Interleukin 10 acts on regulatory T cells to maintain expression of the transcription factor Foxp3 and suppressive function in mice with colitis. Nat Immunol 10: 1178-1184.

33. Glocker EO, Kotlarz D, Boztug K, Gertz EM, Schaffer AA, et al. (2009) Inflammatory bowel disease and mutations affecting the interleukin-10 receptor. N Engl J Med 361: 2033-2045.

34. Brooks AS, Bertoli-Avella AM, Burzynski GM, Breedveld GJ, Osinga J, et al. (2005) Homozygous nonsense mutations in KIAA1279 are associated with malformations of the central and enteric nervous systems. Am J Hum Genet 77: 120-126.

35. Kenwrick S, Watkins A, De Angelis E (2000) Neural cell recognition molecule L1: relating biological complexity to human disease mutations. Hum Mol Genet 9: 879-886.

36. Mariani M, Baldessari D, Francisconi S, Viggiano L, Rocchi M, et al. (1999) Two murine and human homologs of mab-21, a cell fate determination gene involved in Caenorhabditis elegans neural development. Hum Mol Genet 8: 2397-2406.

37. Gupta S, Barrett T, Whitmarsh AJ, Cavanagh J, Sluss HK, et al. (1996) Selective interaction of JNK protein kinase isoforms with transcription factors. EMBO J 15: 2760-2770.

38. Tse W, Zhu W, Chen HS, Cohen A (1995) A novel gene, AF1q, fused to MLL in t(1;11) (q21;q23), is specifically expressed in leukemic and immature hematopoietic cells. Blood 85: 650-656.

39. Garcia-Barcelo MM, Tang CS, Ngan ES, Lui VC, Chen Y, et al. (2009) Genome-wide association study identifies NRG1 as a susceptibility locus for Hirschsprung's disease. Proc Natl Acad Sci U S A 106: 2694-2699.

40. Doray B, Salomon R, Amiel J, Pelet A, Touraine R, et al. (1998) Mutation of the RET ligand, neurturin, supports multigenic inheritance in Hirschsprung disease. Hum Mol Genet 7: 1449-1452.

41. Sugino H, Hamada S, Yasuda R, Tuji A, Matsuda Y, et al. (2000) Genomic organization of the family of CNR cadherin genes in mice and humans. Genomics 63: 75-87.

42. Elson A, Levanon D, Weiss Y, Groner Y (1994) Overexpression of liver-type phosphofructokinase (PFKL) in transgenic-PFKL mice: implication for gene dosage in trisomy 21. Biochem J 299 ( Pt 2): 409-415.

43. Allen PB, Greenfield AT, Svenningsson P, Haspeslagh DC, Greengard P (2004) Phactrs 1-4: A family of protein phosphatase 1 and actin regulatory proteins. Proc Natl Acad Sci U S A 101: 7187-7192.

44. Kim TH, Goodman J, Anderson KV, Niswander L (2007) Phactr4 regulates neural tube and optic fissure closure by controlling PP1-, Rb-, and E2F1-regulated cell-cycle progression. Dev Cell 13: 87-102.

45. Sasaki A, Kanai M, Kijima K, Akaba K, Hashimoto M, et al. (2003) Molecular analysis of congenital central hypoventilation syndrome. Hum Genet 114: 22-26.

46. Garcia-Barcelo M, Sham MH, Lui VC, Chen BL, Ott J, et al. (2003) Association study of PHOX2B as a candidate gene for Hirschsprung's disease. Gut 52: 563-567.

47. Ou-Yang MC, Yang SN, Hsu YM, Ou-Yang MH, Haung HC, et al. (2007) Concomitant existence of total bowel aganglionosis and congenital central hypoventilation syndrome in a neonate with PHOX2B gene mutation. J Pediatr Surg 42: e9-11.

48. Oblinger MM, Wong J, Parysek LM (1989) Axotomy-induced changes in the expression of a type III neuronal intermediate filament gene. J Neurosci 9: 3766-3775.

49. Edery P, Lyonnet S, Mulligan LM, Pelet A, Dow E, et al. (1994) Mutations of the RET proto-oncogene in Hirschsprung's disease. Nature 367: 378-380.

50. Emison ES, McCallion AS, Kashuk CS, Bush RT, Grice E, et al. (2005) A common sex-dependent mutation in a RET enhancer underlies Hirschsprung disease risk. Nature 434: 857-863.

51. Behar O, Golden JA, Mashimo H, Schoen FJ, Fishman MC (1996) Semaphorin III is needed for normal patterning and growth of nerves, bones and heart. Nature 383: 525-528.

52. Stoeckli ET, Lemkin PF, Kuhn TB, Ruegg MA, Heller M, et al. (1989) Identification of proteins secreted from axons of embryonic dorsal-root-ganglia neurons. Eur J Biochem 180: 249-258.

53. Lee MS, Jun DH, Hwang CI, Park SS, Kang JJ, et al. (2006) Selection of neural differentiation-specific genes by comparing profiles of random differentiation. Stem Cells 24: 1946-1955.

54. Moldrich RX, Dauphinot L, Laffaire J, Vitalis T, Herault Y, et al. (2009) Proliferation deficits and gene expression dysregulation in Down's syndrome (Ts1Cje) neural progenitor cells cultured from neurospheres. J Neurosci Res 87: 3143-3152.

55. Le Pecheur M, Bourdon E, Paly E, Farout L, Friguet B, et al. (2005) Oxidized SOD1 alters proteasome activities in vitro and in the cortex of SOD1 overexpressing mice. FEBS Lett 579: 3613-3618.

56. Wynn SL, Fisher RA, Pagel C, Price M, Liu QY, et al. (2000) Organization and conservation of the GART/SON/DONSON locus in mouse and human genomes. Genomics 68: 57-62.

57. Bylund M, Andersson E, Novitch BG, Muhr J (2003) Vertebrate neurogenesis is counteracted by Sox1-3 activity. Nat Neurosci 6: 1162-1168.

58. Southard-Smith EM, Kos L, Pavan WJ (1998) Sox10 mutation disrupts neural crest development in Dom Hirschsprung mouse model. Nat Genet 18: 60-64.

59. Bahn S, Mimmack M, Ryan M, Caldwell MA, Jauniaux E, et al. (2002) Neuronal target genes of the neuron-restrictive silencer factor in neurospheres derived from fetuses with Down's syndrome: a gene expression study. Lancet 359: 310-315.

60. Craxton M (2001) Genomic analysis of synaptotagmin genes. Genomics 77: 43-49.

61. Han J, Yuan P, Yang H, Zhang J, Soh BS, et al. (2010) Tbx3 improves the germ-line competency of induced pluripotent stem cells. Nature 463: 1096-1100.

62. Paulsen FP, Woon CW, Varoga D, Jansen A, Garreis F, et al. (2008) Intestinal trefoil factor/TFF3 promotes re-epithelialization of corneal wounds. J Biol Chem 283: 13418-13427.

63. Mashimo H, Wu DC, Podolsky DK, Fishman MC (1996) Impaired defense of intestinal mucosa in mice lacking intestinal trefoil factor. Science 274: 262-265.

64. Bottner M, Krieglstein K, Unsicker K (2000) The transforming growth factor-betas: structure, signaling, and roles in nervous system development and functions. J Neurochem 75: 2227-2240.

65. Krieglstein K, Strelau J, Schober A, Sullivan A, Unsicker K (2002) TGF-beta and the regulation of neuron survival and death. J Physiol Paris 96: 25-30.

66. Eib DW, Holling TM, Zwijsen A, Dewulf N, de Groot E, et al. (2000) Expression of the follistatin/EGF-containing transmembrane protein M7365 (tomoregulin-1) during mouse development. Mech Dev 97: 167-171.

67. Tsukahara F, Hattori M, Muraki T, Sakaki Y (1996) Identification and cloning of a novel cDNA belonging to tetratricopeptide repeat gene family from Down syndrome-critical region 21q22.2. J Biochem 120: 820-827.

68. Mongroo PS, Rustgi AK (2010) The role of the miR-200 family in epithelial-mesenchymal transition. Cancer Biol Ther 10: 219-222.

69. Sakurai M, Ayukawa K, Setsuie R, Nishikawa K, Hara Y, et al. (2006) Ubiquitin C-terminal hydrolase L1 regulates the morphology of neural progenitor cells and modulates their differentiation. J Cell Sci 119: 162-171.

70. Larsson LT (1994) Hirschsprung's disease--immunohistochemical findings. Histol Histopathol 9: 615-629.

71. Wakamatsu N, Yamada Y, Yamada K, Ono T, Nomura N, et al. (2001) Mutations in SIP1, encoding Smad interacting protein-1, cause a form of Hirschsprung disease. Nat Genet 27: 369-370.

72. Amiel J, Espinosa-Parrilla Y, Steffann J, Gosset P, Pelet A, et al. (2001) Large-scale deletions and SMADIP1 truncating mutations in syndromic Hirschsprung disease with involvement of midline structures. Am J Hum Genet 69: 1370-1377.

73. Van de Putte T, Francis A, Nelles L, van Grunsven LA, Huylebroeck D (2007) Neural crest-specific removal of Zfhx1b in mouse leads to a wide range of neurocristopathies reminiscent of Mowat-Wilson syndrome. Hum Mol Genet 16: 1423-1436.

74. Nagai T, Aruga J, Takada S, Gunther T, Sporle R, et al. (1997) The expression of the mouse Zic1, Zic2, and Zic3 gene suggests an essential role for Zic genes in body pattern formation. Dev Biol 182: 299-313.

75. Sanchez-Camacho C, Bovolenta P (2008) Autonomous and non-autonomous Shh signalling mediate the in vivo growth and guidance of mouse retinal ganglion cell axons. Development 135: 3531-3541.
